# Disentangling geographical, biotic, and abiotic drivers of plant diversity in neotropical *Ruellia* (Acanthaceae)

**DOI:** 10.1371/journal.pone.0176021

**Published:** 2017-05-04

**Authors:** Erin A. Tripp, Yi-Hsin Erica Tsai

**Affiliations:** 1 Department of Ecology and Evolutionary Biology, University of Colorado, Boulder, Colorado, United States of America; 2 Museum of Natural History, University of Colorado, Boulder, Colorado, United States of America; Chinese Academy of Forestry, CHINA

## Abstract

It has long been hypothesized that biotic interactions are important drivers of biodiversity evolution, yet such interactions have been relatively less studied than abiotic factors owing to the inherent complexity in and the number of types of such interactions. Amongst the most prominent of biotic interactions worldwide are those between plants and pollinators. In the Neotropics, the most biodiverse region on Earth, hummingbird and bee pollination have contributed substantially to plant fitness. Using comparative methods, we test the macroevolutionary consequences of bird and bee pollination within a species rich lineage of flowering plants: *Ruellia*. We additionally explore impacts of species occupancy of ever-wet rainforests vs. dry ecosystems including cerrado and seasonally dry tropical forests. We compared outcomes based on two different methods of model selection: a traditional approach that utilizes a series of transitive likelihood ratio tests as well as a weighted model averaging approach. Analyses yield evidence for increased net diversification rates among Neotropical *Ruellia* (compared to Paleotropical lineages) as well as among hummingbird-adapted species. In contrast, we recovered no evidence of higher diversification rates among either bee- or non-bee-adapted lineages and no evidence for higher rates among wet or dry habitat lineages. Understanding fully the factors that have contributed to biases in biodiversity across the planet will ultimately depend upon incorporating knowledge of biotic interactions as well as connecting microevolutionary processes to macroevolutionary patterns.

## Introduction

Repeated observations in unrelated families of greater species richness in the Neotropics compared to the Paleotropics has yielded a now classic pattern in plant biogeography [1–2]. In recent decades, several studies have sought possible mechanisms to explain this pattern and one emerging consensus is that of higher net diversification rates in the Neotropics compared to Paleotropics [3]. This finding has been attributed to a variety factors including (a) aridification since the Miocene resulting in more severe contraction of rainforests and higher extinction in the Paleotropics compared to Neotropics and (b) uplift of the Andes in the Neogene followed by opportunistic niche occupation in the Neotropics [1–5]. Yet, current knowledge of this topic remains limited by past emphasis on abiotic drivers of diversification at the expense of biotic drivers. Biotic interactions are assumed to be as important to biodiversity evolution but are less commonly studied owing to inherent complexity and numbers of such interactions [6–8].

Amongst the most prominent of biotic interactions are those between plants and pollinators. Hummingbirds, bees, butterflies, flies, moths, bats, and other animals are obligate symbionts of tens of thousands of flowering plants and play prominent roles in plant speciation [9–12]. Pollinator-driven diversification was first conceptualized by Darwin [13] and later codified by 20^th^ Century authors [14–16]. Under this model, animal pollinators act as drivers of floral divergence, which may then complement additional reproductive isolating mechanisms during speciation [17]

Hummingbird pollination in particular contributes substantially to plant fitness in the Neotropics and has been the focus of recent research [18–19]. On the one hand, hummingbird pollination has been hypothesized to drive plant diversification. For example, Bradshaw & Schemske [20] demonstrated that single locus mutations can give rise to major floral innovations that facilitate rapid divergence in pollination system and Temeles & Kress [21] demonstrated intricate matching of floral morphologies and hummingbird bills. Kay [17] further showed specialized pollination by hummingbirds to be a primary mechanism for reproductive isolation, and Schmidt-Lebuhn et al. [22] documented numerous lineages of flowering plants in which numbers of species in hummingbird-pollinated clades substantially outweigh numbers of species in insect-pollinated sister clades. On the other hand, hummingbird pollination could yield lower rates of plant speciation when one considers other ecological processes. For example, birds are in general capable of traveling greater distances than insect pollinators thereby having greater capacity to maintain genetic connectivity among spatially distant plant populations. In this manner, population level processes such as patterns of gene flow should scale up to and help predict major patterns in macroevolution.

**Fig 1:**
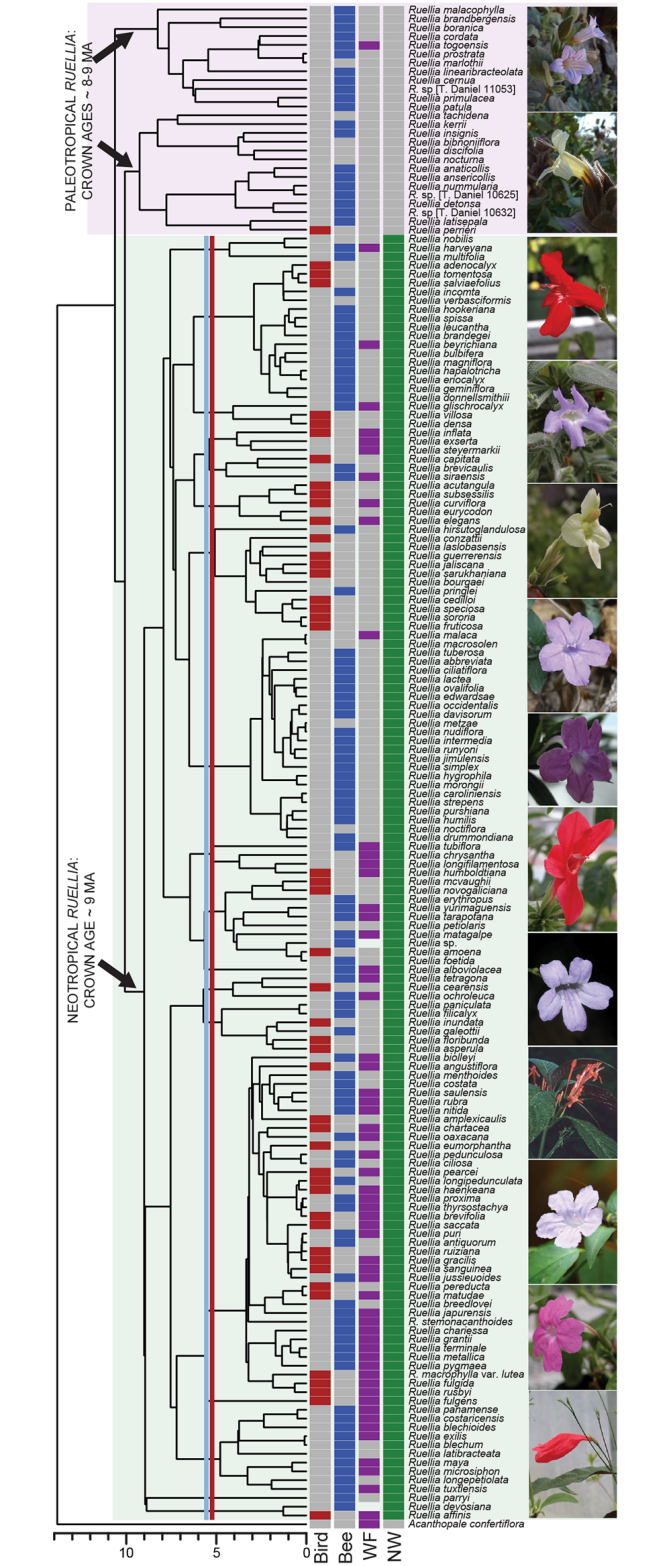
Maximum clade credibility phylogeny for relationships among species of *Ruellia*. Colored boxes show each taxon’s character states: bird-adapted (red), bee-adapted (blue), wet forest inhabiting (purple), and New World residency (green). The two vertical lines demarcate the crown age for the oldest hummingbird (*R*. *fulgens*; Guiana Shield; red) and the oldest bee (*R*. *alboviolacea*; Mexico; blue) lineage among Neotropical *Ruellia*; both date to ~5.8 Ma. Photos are examples of bird (red, yellow, and pink-flowered) and bee-adapted (purple flowered) species of *Ruellia*. From top to bottom: *R*. *patula*, *R*. *insignis*, *R*. *elegans*, *R*. *galeottii*, *R*. *speciosa*, *R*. *lantanoglandulosa*, *R*. *maya*, *R*. *affinis*, *R*. *pittieri*, *R*. *haenkeana*, *R*. *matudae*, *R*. *pearcei*. Phylogeny is reprinted from Tripp & McDade (2014) under a CC BY license, with permission from *Aliso*, original copyright in 2014.

Following the above, we hypothesize that discrepancies in flowering plant diversity between the Neotropics and Paleotropics may in part be accounted for by the presence of hummingbird pollination in the former, but not the latter [18]. If true, it may be expected that hummingbird-pollinated lineages have higher speciation rates than non-hummingbird pollinated lineages. However, high Neotropical diversity vis-a-vis Paleotropical diversity is unlikely to be explained by a single factor [23–24]. As such, we additionally attempted to disentangle the effects of other potential drivers of high Neotropical diversity including historical and abiotic factors. Our focal lineage is the geographically widespread and ecologically important genus *Ruellia* (Acanthaceae), which contains upwards of 400 extant species (Fig 1). Species diversity in *Ruellia* is concentrated in a monophyletic Neotropical lineage, which is derived from a grade of Paleotropical lineages. On the one hand, approximately half of all sister species pairs in this Neotropical clade have divergent pollination systems, unlike Paleotropical. On the other hand, aridification throughout the Neogeone had substantial impacts on plant diversification throughout the Neotropics [25], and lineage habitat shifts between contrasting tropical biomes such as ever-wet and dry habitats are highly characteristic of Neotropical *Ruellia* [26]. In contrast, Paleotropical *Ruellia* are far more homogenous in pollination system (typically, bee or hawkmoth) and habitat (typically, dry habitat [27]). Using comparative phylogenetic methods, we first tested our assumption of a difference in net diversification rates between (1) Paleotropical and Neotropical *Ruellia*. After confirming higher rates in the latter, we then tested differences in net diversification rates between (2) hummingbird-adapted vs. non-hummingbird lineages, (3) bee-adapted vs. non-bee adapted lineages, and (4) wet vs. dry habitat lineages. A positive association between diversification rates and pollination mode and/or habitat shifts would implicate biotic and/or abiotic factors contributing to increased diversification in the Neotropics compared to the Paleotropics. Failure to detect an association between diversification rate and any characters may instead suggest that simply the opening of new habitats in the Neotropics following a single dispersal event from the Paleotropics, and subsequent filling of newly available niches, may have been sufficient to spur diversification in Neotropical *Ruellia*.

## Materials and methods

### Taxon sampling & character matrix preparation

Of ~400 species of *Ruellia*, ~300 occur in the Neotropics and the remaining ~100 are Paleotropical (Tripp & Darbyshire, in press). This study sampled 173 total taxa: 172 species of *Ruellia* (plus one outgroup, *Acanthopale confertiflora*) spanning the taxonomic, morphological, ecological, and geographical variation present within the genus [26]. Of these 172, 146 are Neotropical and 26 are Paleotropical, thus our taxon sampling approximates the ratio of Neotropical to Paleotropical species. To enable testing of the above competing but not necessarily mutually exclusive drivers of diversification including biotic and abiotic factors, we implemented a stepwise workflow as follows, testing whether there was a diversification rate difference between: (1) Paleotropical vs. Neotropical members of *Ruellia*, (2) hummingbird-adapted vs. non-hummingbird-adapted lineages, (3) bee-adapted vs. non-bee-adapted lineages, and (4) lineages inhabiting wet vs. dry habitats. Character states scored in this study (see below) along with examples of floral morphological diversity are shown in Fig 1.

The 172 species of *Ruellia* comprise several distinct classes of pollination systems (i.e., functional groups) sensu Tripp & Manos [11]: bee, butterfly, hawkmoth, bat, and bird-adapted species. Prior work including extensive field study has confirmed a match between these functional groups of pollinators and plant pollination syndromes in *Ruellia* [11], lending these data to large-scale comparative investigation of pollinator driven diversification. In this study, we focused on hummingbird- and bee- adapted species because prior research has demonstrated that rare states pose challenges to diversification analyses [28]. Whereas hummingbird-adapted (n = 46) and bee-adapted (n = 108) species comprise ~91% of the dataset, species adapted to bat, butterfly, or hawkmoth pollination comprise <10% of the dataset (n = 18 total). Our sampling of hummingbird-adapted and bee-adapted species similarly approximates the full ratio of these two states across Neotropical *Ruellia*, although we note that the genus has yet to be fully revised.

We classified the 172 *Ruellia* as occupying either wet or dry habitat (no species sampled in this study spans both categories). Here, wet habitats are taken to be those that remain ever-wet year-round and lack a pronounced dry season; they are primarily forested. Dry habitats are taken to be those that marked by a strong dry season and, if forested, experience a deciduous or subdeciduous event annually. Dry habitats here encompass a broad variety of ecosystems ranging from forested environments (e.g., "selva baja caducifolia" and "selva mediana subcaducifolia/subperennifolia" in Mexico; caatinga in Brazil; Chiquitano in Bolivia and Brazil; Chaco in Argentina) to savannas (e.g., Gran Sabana in Venezuela; cerrado in Brazil; pampas in Argentina). We were unable to use mean annual precipitation as an alternative means of delimiting wet vs. dry forests because direct estimates of this variable are lacking in numerous regions of the Neotropics that species of *Ruellia* inhabit. In this study, 59 species were scored as belonging to wet habitats vs. 113 scored as belonging to dry habitats. None of the species included in our dataset were polymorphic for habitat type.

### Phylogenetic & diversification analyses

Our study used the time-calibrated maximum clade credibility (MCC) tree presented in Tripp & McDade [18], pruned to contain only ingroup taxa plus one outgroup. To test for associations between plant lineage diversification rate and pollination system, we undertook a series of trait-based diversification analyses using the R package diversitree v.0.9–7 [29]. These diversification models have come under recent criticism because of their uncertain performance when dealing with incomplete taxon sampling as well as rare traits, their tendency towards high Type I and Type II error rates, and the potential for spurious correlations for what are in effect neutrally evolving characters [29–34] (but see [30–31]). The present study addresses these shortcomings through several approaches. First, the dataset herein employed represents the densest taxon sampling yet achieved with which to explore plant-pollinator diversification, and we focus on the two most common character states thereby avoiding problems associated with rare traits. Second, whereas prior trait-based diversification studies have followed a traditional hypothesis-testing approach where likelihood ratio tests are used to assess model fits against one another [28, 34], we here utilize an additional, alternative approach to model selection that improves parameter estimation through weighted model averaging rather than selecting one best fitting model, which is known to suffer from robustness [33]. Third, we focus on one of the most important classes of plant traits to plant fitness: pollination mode. Although not immune to spurious correlations in comparative analyses, mode of pollination is in most cases under strong selection rather than neutrally evolving, thus minimizing the likelihood of a completely spurious correlation. We implemented diversification rate analyses using the BiSSE modeling framework rather than the MuSSE framework because BiSSE models have been studied more extensively and their error rates are better understood [31].

The diversification models evaluated varied in complexity, ranging from allowing all rates to vary independently for each character state (i.e., having 6 free parameters: λ_0_, λ_1_, μ_0_, μ_1_, q_01_, q_10_, where 0 and 1 refer to the absence or presence of the pollination system) to fixing rates to be equal between the character states (i.e., having only 3 free parameters where λ_0_ = λ_1_, μ_0_ = μ_1_, and q_01_ = q_10_) (Table 1). We explored impacts of phylogenetic uncertainty by repeating diversification rate analyses of the bird-adapted and bee-adapted datasets on 100 randomly chosen trees from a Bayesian posterior distribution (presented in [18]); results were consistent with tests conducted on the MCC tree and thus only results from the latter are presented.

**Table 1:** Diversification models used to understand the evolution of pollination syndromes (bird and bee), habitat shifts (wet or seasonally dry forests), and transitions across continents (old world to new world) in *Ruellia*. λ = speciation rate; μ = extinction rate; q = transition rate. State 1 is for bird or bee pollinated, wet forest, and new world; state 0 is non-bird or non-bee pollinated, seasonally dry forest, and old world. In bold are the lnLik of the best models according to likelihood ratio tests and wAIC scores greater than 0.1.

Model Name	6	5A	5B	5C	4A	4B	4C	3
Parameters	λ0, λ1, μ0, μ1, q01, q10	λ0 = λ1, μ0, μ1, q01, q10	λ0, λ1, μ0 = μ1, q01, q10	λ0, λ1, μ0, μ1, q01 = q10	λ0 = λ1, μ0 = μ1, q01, q10	λ0 = λ1, μ0, μ1, q01 = q10	λ0, λ1, μ0 = μ1, q01 = q10	λ0 = λ1, μ0 = μ1, q01 = q10
No. parameters	6	5	5	5	4	4	4	3
lnLik								
OW NW	-364.5	-369.0	-364.5	-364.7	-373.4	-369.0	**-364.7**	-373.6
Bird	-435.5	-438.9	**-435.7**	-447.4	-440.9	-451.1	-451.8	-452.8
Bee	-465.0	-465.9	-465.0	-466.9	**-465.9**	-467.5	-467.0	-468.3
Habitat	-451.3	-451.7	-451.3	-456.2	**-452.0**	-456.5	-456.6	-456.7
wAIC								
OW NW	0.08	0.00	**0.22**	**0.18**	0.00	0.01	**0.50**	0.00
Bird	**0.29**	0.03	**0.68**	0.00	0.01	0.00	0.00	0.00
Bee	0.09	**0.11**	**0.26**	0.04	**0.28**	0.06	0.09	0.07
Habitat	**0.11**	**0.20**	**0.28**	0.00	**0.40**	0.00	0.00	0.01

We implemented two different approaches to model fitting to produce parameter estimates as well as investigate the effects of different model fitting strategies on results. First, we followed a traditional model selection approach that utilized a series of transitive likelihood ratio tests to select best fitting models. Second, we implemented a weighted model averaging approach [35] that is here for the first time applied to diversification analyses. This method averages across multiple high fitting models rather than selecting one best fitting model then relying solely on parameter estimates from that best fit model. To calculate model weights, we first estimated maximum likelihoods for each model using diversitree [29]. We then ranked all models according to the resulting AIC scores and calculated weighted AIC scores. The marginal distributions for each parameter were then combined following the wAIC scores (model weights) for each of the 8 models. Speciation, extinction, and transition rates between the two trait classes were compared by assessing the amount of overlap area between the marginal distributions for each parameter pair. The data matrix associated with analyses has been included in the Supporting Information of this paper (S1 Dataset).

### Diversitree implementation details

Because the find.mle function in diversitree is highly sensitive to starting point, our maximum likelihood analyses were repeated up to 729 times with starting points systematically drawn from a starting point matrix. The starting point matrix for each dataset was established from two pilot MLE analyses based on the most parameter-rich model: one that used a starting point drawn from a uniform distribution between 0 and 1 for each parameter and a second that used a starting point based on the character-independent birth-death model implemented via the starting.point.bisse function in diversitree (24). Parameter estimates chosen to populate the starting point matrix derived from the pilot analysis with the higher MLE score. The starting point matrix contained three values for each parameter: the best MLE, the MLE * 10 and the MLE * 0.1. Maximum likelihood analyses were then conducted based on all combinations of possible parameter starting points from the matrix. The highest MLE derived from the runs was used to calculate the weighted contribution of that model to the weighted average model.

Posterior distributions were estimated for each parameter using the Bayesian mcmc function in diversitree for each model and dataset [29]. The highest MLE derived from all maximum likelihood analyses was used as the starting point. We applied an exponential distribution as the prior for each parameter with rate 1/(2r), where r is the character independent diversification rate scaled to the length of the MCC tree. The tuning parameter vector (w) was chosen based on a short pilot study of each model to improve run times. Full mcmc analyses were run for 10,000 steps, and marginal distributions of each parameter were combined across models proportional to the model’s wAIC score (with a precision of 0.01) to produce weighted averaged posterior distributions.

## Results

**Fig 2:**
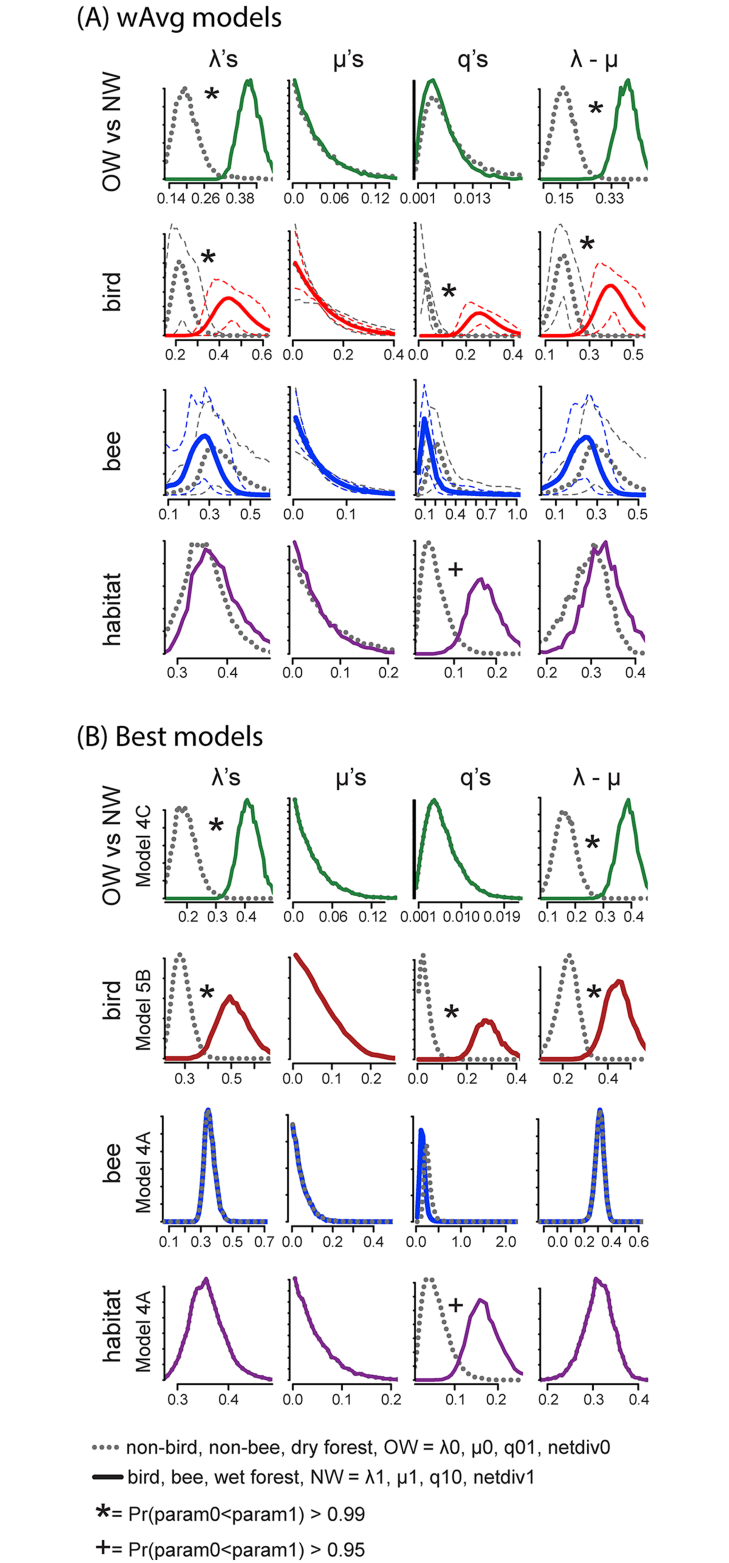
Rates of evolution of different trait classes among species of *Ruellia*. Speciation (λ), extinction (μ), transition (q), and net diversification (λ-μ) rate distributions are shown for each trait. (A) Parameter distributions from the weighted average of all the models tested for each dataset. (B) Parameter distributions of only the best model for each dataset. All models are shown in S1–S4 Figs. The bird and bee datasets were rerun on 100 randomly chosen trees from the Bayesian posterior distribution. The 90% confidence intervals resulting from those runs are shown via dashed lines with corresponding colors.

Our first objective was to test the assumption that Neotropical *Ruellia* in fact is marked by a higher diversification rate than Paleotropical *Ruellia*. We found broad support for this assumption: top fitting individual models (i.e., 4C, 5B, 5C, 6) as well as the weighted average model yielded evidence for higher net diversification among Neotropical *Ruellia* (Fig 2 and S1 Fig; Table 1; S1–S3 Tables). The weighted model suggests this pattern is driven by a higher speciation rate within the Neotropical clade rather than differences in extinction rates (Table 1; S1–S3 Tables). We found no differences in transition rates between the two groups (Fig 2 and S1 Fig; Table 1; S1–S3 Tables), which was expected given a single transition from the Paleotropics to the Neotropics in our dataset.

We then explored potential impacts of hummingbird pollination as well as bee pollination on diversification rates. We found support for the hypothesis that hummingbird pollination may be associated with increased diversification rates, both under the top-fitting model (i.e., model 5B) and the overall weighted average; this result was strongly supported under models allowing independent speciation rates for hummingbird-adapted and non-hummingbird-adapted lineages (i.e., 5B, 6), which accounted for 96% of the weighted average model (Fig 2 and S2 Fig, Table 1; S1–S3 Tables). On the other hand, bee-adapted lineages did not show higher speciation, extinction, or net diversification rates in either the best model (i.e., 4A) or the weighted average, but rather only in some sub-optimal fitting models (S3 Fig, Table 1; S1–S3 Tables). Our data also showed that a loss of bird pollination is faster than its gain, but no transition rate differences with respect to bee-adapted lineages (Fig 2, S2 and S3 Figs; Table 1; S1–S3 Tables).

We tested whether occupancy of a given habitat may have helped drive high diversification in *Ruellia*. We recovered no support for an association between either wet or dry habitats type and net diversification rate (Fig 2 and S4 Fig; Table 1; S1–S3 Tables). In the top-fitting model (i.e., 4A) as well as the weighted average, probability curves were nearly overlapping between wet forest vs. dry forest lineages for speciation, extinction, and net diversification (Fig 2 and S4 Fig; Table 1; S1–S3 Tables). However, we found borderline significant differences (Pr > 0.988) in transition rates, with a trend towards higher rates of loss of wet forest habitat than gain; all models with multiple transition rate parameters had non-negligible contributions to the overall weighted average model (Fig 2 and S4 Fig; Table 1; S1–S3 Tables).

## Discussion

The Neotropics represent the most biodiverse region on Earth per unit area and thus serves as one of the most suitable *in situ* laboratories for understanding the relationship between biotic interactions, abiotic interactions, and speciation [36]. In Acanthaceae specifically (~ the 10th most diverse family of flowering plants), *Ruellia* is only one of numerous lineages marked by a pattern of greater species richness in the Neotropics compared to Paleotropics (Table 2; and [37–41]). The *Ruellia* dataset herein utilized is unique among comparable studies in other flowering plant families because of the large number of sampled Neotropical species marked by exceptionally high diversity in pollinator and habitat transitions [11, 25]. Our study provides empirical evidence for increased net diversification rates associated with hummingbird-adapted lineages of Neotropical plants but no evidence for diversification rate differences between bee- or non-bee-adapted lineages as well as no rate differences between wet vs. dry forest plant lineages. However, robustness of the above patterns was dependent on method of model selection or model averaging, reiterating the importance of model selection in phylogenetic comparative analyses [35] We fit data to 8 fully nested models that incorporate speciation (λ), extinction (μ), and transition (q) rates, then compared results from the top fitting model to the weighted model derived from averaging parameter distributions. Whereas the top-fitting model yielded the strongest evidence for increased diversification rates, this signal was diminished in the weighted average model.

**Table 2:** Repeated instances in which standing taxonomic diversity in the Neotropics far exceeds standing diversity in the Paleotropics, per given monophyletic lineage within Acanthaceae. The # of species column refers to the number sampled or studied in the references cited column rather than the actual number of extant species in this lineage.

Clade Name	# Species (Total)	# Species (Neotropics)	# Species (Paleotropics)	Reference
Acantheae	286	269	17	34
Isoglossinae	116	92	24	35
*Mendoncia*	*57*	54	3	36
*Justicia*		500	200	37
*Ruellia*	400	300	100	This study; 27
*Tetramerium* lineage	170	125	45	38

### Contributions to the Neotropical biodiversity debate

Comparison of hummingbird- and bee-adapted lineages within a single, species-rich monophyletic genus of angiosperms provides a direct test of evolutionary rate differences associated with these two different pollination modes. Results from this study point to higher diversification rates for hummingbird-adapted but not bee-adapted lineages, the former of which can be attributed to higher speciation rates. Although not explicitly tested in this study owing to too few data points, hawkmoth- and bat-adapted lineages (these here incorporated in the "non-bird" or "non-bee" character states) are likely to be associated with lower diversification rates. One potential explanation for this discrepancy in standing diversity between hummingbird- or bee- vs. hawkmoth or bat-adapted species relates to the evolutionary fates of these various pollination systems in *Ruellia*: whereas both hummingbird- and bee-adapted species can and do give rise to new evolutionary lineages with different pollination systems, hawkmoth and bat-adapted lineages are evolutionary dead-ends (i.e., rarely to never giving rise to lineages with different pollination systems [11]). In the present study, we found faster evolutionary rate losses of hummingbird pollination compared to gains, which may simply reflect relative probabilities of potential hummingbird pollinators vs. other pollinators: only 330 species of hummingbirds exist on Earth [18] compared to tens of thousands of pollinating bees and other insects [42].

In contrast, no support was recovered for an association between higher diversification rates and occupancy of wet or dry habitats. Given that species of *Ruellia* are clearly marked by high diversity in habitat, our *a priori* expectation was that habitat switching has played a role in diversification of *Ruellia*. In particular, Acanthaceae are well documented to be especially diverse and abundant in dry or arid environments. In *Ruellia*, cerrado and campos rupestres habitats typical of Bahía and Goiás, Brazil, host extremely high levels of diversity and endemism in *Ruellia* (ca. 60 species); similarly, seasonally dry semi-deciduous forests of the Sierra Madre del Sur in southern Mexico (ca. 60 species) as well as dry forests of Madagascar (ca. 40 species) represent other major centers of diversity for the genus (Tripp, unpub data). Other lineages of Acanthaceae follow similar trends. Several genera including *Petalidium*, *Blepharis*, *Barleria*, and *Monechma* are incredibly diverse and comprise vast portions of total vegetative cover in Namibia, which is the driest country in the southern hemisphere (Tripp et al., in review). In our study, it is plausible that frequent switches among habitats rather than occupancy of and subsequent diversification within a given habitat was a driving factor in speciation of this group. Alternatively, given the diversity of dry habitats exploited by *Ruellia*, it is possible that signatures of increased diversification may be recovered if we parsed these habitats into more narrowly defined ecosytems. That is, we here treated all 'dry' habitats under one character state even though drylands comprising the Neotropics are extraordinarily diverse and range from seasonally dry tropical forests to savannas to chaco vegetation, and so forth [43]. This parsing may in particular yield evidence for increased diversification rates in areas marked by high extant species diversity such as Mexican dry forests or Brazilian caatinga + cerrado [44]. Such an analysis would benefit from a near complete species-level sampling of Neotropical *Ruellia* (Tripp et al., in prep.). Although not significant in our analyses, there is a slight trend towards faster rates of loss of wet forest habitat than gains. This may in part be attributable to the increase in seasonally dry tropical forests and other dryland ecosystems such as savannas throughout the Miocene [44], when *Ruellia* was undergoing diversification [18]. Nonetheless, based on present taxon sampling and method of analysis, neither wet nor dry habitats help to explain high Neotropical diversity in this genus.

In this study, we did not explicitly conduct any tests of correlation between species richness and clade age [45–46] or between species richness and the ages of origins of dry habitats. However, crown ages of both Neotropical and Paleotropical *Ruellia* date to ~9 Ma (Fig 1). As such, clade age alone does not seem to be a viable predictor variable of differential species richness between Paleotropical and Neotropical *Ruellia*. Additionally, among Neotropical *Ruellia*, extant lineages containing hummingbird- or bee-adapted species both date to ~5.8 Ma (crown ages; Fig 1) suggesting that standing diversity of *Ruellia* species with different modes of pollination and time are likely decoupled [45]. Finally, there is ample evidence that ever-wet Neotropical lowland rainforests became established and diversified somewhere near the Paleocene-Eocene Thermal Maximum (ca. 55 MY before present) or slightly before [46–48]. In contrast, the onset of widespread drying of the Neotropics and subsequent origins of shrublands, grasslands, and seasonally dry tropical forests is much more recent (<15 MY before present [47]). Thus, depending on the extent of dry ecosystems when *Ruellia* first began to diversify, wet forest lineages may have had more time to accumulate species diversity than dry ecosystem lineages. Yet, dry-adapted lineages are over two times as species rich based on the present sampling (Fig 1). Given that we failed to detect a relationship between dry habitats and increased diversification rates in the present study, it seems plausible that higher extinction rates may characterize lineages in wet ecosystems and that the problem of estimating extinction rates continues to plague comparative studies, as in the present analysis [49].

Taken together, our data suggest that biotic factors—specifically adaptation to hummingbird-pollination—may help explain high plant diversification in the Neotropics [3]. Hummingbirds are present today in the Neotropics but not the Paleotropics, thus adding an additional biotic driver to one hemisphere but not the other. Hummingbirds were however once known from the Old World prior to going extinct there [18], but there is no evidence that they were either diverse or widespread in that region; in fact, all Old World hummingbird fossils recovered to date derive from a relatively small geographical area that includes Germany and France [18, 48–49]. Given an origin of hummingbird pollination in the Neotropics that long predates the dispersal and origin of the *Ruellia* clade there [18], it seems clear that these pollinators set an important ecological stage upon which plants diversified (rather than an alternative of contemporaneous, co-evolutionary scenarios of diversification; [18–19, 50]. In sum, full understanding of global biodiversity hotspots such as the Neotropics may be muddled by primary emphasis on abiotic variables at the expense of biotic variables, and the inherent biotic interaction that exists between plants and obligate animal pollinators provides a means of assessing the importance of biotic interactions.

### Model averaging vs. model selection

The traditional model selection approach utilizes a series of transitive likelihood ratio tests to select the model of best fit. In the present study, this traditional approach yielded similar conclusions to those deriving from our model averaging approach. However, this is unlikely to be the case in all or even most empirical studies. Datasets with moderate levels of conflict in the data are likely to yield different results depending on whether a best-model or a weighted average approach is used. Our view is that the model averaging approach yields a more nuanced perspective on diversification patterns, one that can be harder to interpret and less definitive, but may sometimes more closely reflect true evolutionary history. This tempering of results from BISSE may answer criticisms that diversitree overestimates its confidence in identifying traits as diversification drivers (see recent, thoughtful perspectives in [30–31]). As a corollary, our results for bird pollination are supported by both methods, improving confidence in the robustness of our results.

### Plant-pollinator interactions and macroevolution: Is there a consensus?

Despite the importance of pollinator driven diversification [51], the relationship between plant lineage species richness and pollinator functional group has remained poorly studied compared to the influences of other traits. This deficit is especially striking in the tropics given the tremendous diversity of both plants and pollinators that these ecosystems support. In Paleotropical or Paleo-subtropical regions such as the Cape Floristic Province, research on some of the most diverse groups of plants (e.g., *Protea*, *Moraea*, *Babiana*) has demonstrated a relative lack of effect of pollination system or floral traits on diversification rates [52]. Similarly in the Cape Floristic Province, Forest et al. [53] documented no differences in rates of diversification among clades of *Lapeirousia* (Iris Family) that are pollinated by different animal functional groups. In contrast, Valente et al. [54] found that diversification rates were higher in Cape Floristic Province plant lineages with a higher diversity of pollination systems vs. those with a single pollination system. In two species-rich clades of Australian legumes, Toon et al. [55] found lower rates of diversification in old world bird-pollinated than in bee-pollinated lineages. In the Neotropics, there exist fewer studies that have addressed macroevolutionary consequences of pollinator functional group on plant lineage species richness. In several large lineages of orchids (mixed Neotropical and Paleotropical taxon sampling), Schiestl & Schlüter [56] found no correlation between pollination system and plant diversification. Similarly, in the large genus *Dalechampia* (also mixed Neotropical and Paleotropical taxon sampling), Armbruster et al. [57] documented no effect of pollination system on diversification rates. In Cactaceae, Hernández-Hernández et al. [58] found that shifts to derived pollination systems such as bat, bird, and nocturnal moths were associated with higher rates of plant diversification. In one of the most compelling studies to date, Roalson & Roberts [24] found strong evidence for higher diversification rates in two clades of Gesneriaceae pollinated primarily by hummingbirds.

The above showcases a lack of consensus yet to emerge from plant-pollinator diversification studies. However, we emphasize that only a very small fraction of flowering plants lineages has, to date, been included in such studies, and a better consensus awaits inclusion of data from many more lineages of plants as well as bringing data from ecological and microevolutionary studies to bear on the subject (see below). A better consensus should also include parallel studies from the hummingbird perspective. For example, studies of the patterns of bird diversification can similarly shed light on the importance of biotic interaction, and complementary ecological approaches are necessary to understand *how* plant diversity and abundance impact bird diversity and abundance at a local scale. Nonetheless, our results are consistent with a biotic interactions hypothesis wherein competitive or beneficial relationships among species represent a “paramount adaptive problem” ([59]) and become primary drivers of speciation.

### Where do we go from here? Linking ecological processes to macroevolutionary patterns

In 1982, Gentry puzzled over what he called an excess of plant species diversity in the Neotropics compared to the Paleotropics [1]. Antonelli et al. [3] recovered evidence for Neotropical ecosystems acting as an “engine for global plant diversity”. Are these patterns of diversity explainable by fine scale biotic processes operating on multiple spatial and temporal levels? Macroevolutionary predictions suggest that the full assemblage of plant traits associated with hummingbird pollination may either promote or decrease net diversification rates. For example, whereas features such as specialized behavior by pollinators, high pollen transfer precision, dispersal limitation of pollen, and better floral rewards and cues may in some cases promote reproductive isolation and lead to higher diversification rates among hummingbird-adapted plant lineages, larger travel distances possible by hummingbird pollinators and higher plant extinction rates associated with specialized ecologies may yield lower diversification rates among hummingbird-adapted plant lineages. Despite the tremendous importance of pollinator-driven diversification in understanding correlates of biodiversity, a conceptual framework for linking pollination system with patterns of diversification is lacking beyond expectations derived from general models such as isolation by distance [60–62]. The above ambiguity highlights our lack of a robust theoretical framework for how and under what contexts microevolution scales up to explain macroevolution. In sum, understanding fully the factors that have contributed most strongly to biases in biodiversity across the planet will ultimately depend upon our capacity to link microevolutionary processes to macroevolutionary patterns.

## Conclusions

Although a full explanation for higher Neotropical biodiversity *vis-à-vis* temperate and other ecosystems is certain to include a panoply of explanations ranging from historical contingency (e.g., accumulated diversity and escape from recent glaciation events of mass destruction) to thermal kinetics, an abundance of data support the biotic interactions hypothesis [63–67]. Our study provides some of the first evidence consistent with a hypothesis of a connection between hummingbird pollination and species richness in Neotropical plants. The idea that hummingbird pollination specifically is correlated to high plant diversity has been recognized for some time but has rarely been tested rigorously using robust datasets and likelihood-based comparative methods. Support for this hypothesis rests in part on the notion that hummingbirds are more efficient at transporting and delivering plant pollen than are other animal functional groups, thus leading to repeated gains of hummingbird pollination across thousands of plant lineages in the Neotropics that are well documented empirically. In addition to data presented here, this hypothesis is also supported by previous findings that hummingbird pollination is not an evolutionary dead-end.

Nonetheless, the present lack of consensus regarding the impacts of pollination systems on plant diversification may be attributable to a paucity of research linking microevolutionary to macroevolutionary phenomena. The scaling of population-level processes to explain broader evolutionary phenomena in plant-animal interactions thus offers a potent area for future empirical and synthetic research in the field. Forward progress on such important debates will need to consider not just abiotic or biotic variables in isolation, but interactions between the two, for example plant adaptations to wet habitats or the evolution of perennial life histories, both of which help set the stage for biotic interactions such as that between plants and hummingbirds [68].

## Supporting information

S1 Dataset Matrix of all character trait data used in the diversification rate analyses of *Ruellia*. Characters scored for each taxon are: bird (1) /non-bird (0) pollinated, bee (1) /non-bee (0) pollinated, wet forest (1) /dry forest (0) inhabiting, and New World (1) /Old World (0) speices.
(CSV)

S1 Fig Marginal distributions of all parameters for each model comparing rates of evolution in Paleotropical vs. Neotropical *Ruellia*. All models including the weighted average model are shown.
(TIF)

S2 Fig Marginal distributions of all parameters for each model of the evolution of hummingbird pollination in *Ruellia*. All models including the weighted average model are shown. The best model according to likelihood ratio tests is in bold.
(TIF)

S3 Fig Marginal distributions of all parameters for each model of the evolution of bee pollination in *Ruellia*. All models including the weighted average model are shown. The best model according to likelihood ratio tests is in bold.
(TIF)

S4 Fig Marginal distributions of all parameters for each model of the evolution of habitat type in *Ruellia*. All models including the weighted average model are shown. The best model according to likelihood ratio tests is in bold.
(TIF)

S1 Table Diversification models and marginal parameter estimates used to understand the evolution of bee and bird pollination syndromes, habitat shifts, and transitions across continents (Old world to new world) in *Ruellia*. Models are defined in Table 1. Median parameter estimates are reported with 5th and 95th percentiles given in parentheses. The best models are in bold.
(DOCX)

S2 Table Probabilities that the marginal distribution of one parameter is less than another parameter. Models are defined in Table 1. Asterisks indicate significant values either less than 0.05 or greater than 0.95. NA’s indicate comparisons of parameters that were set to be equal in the model. The best models are shown in bold. For analyses averaged over 100 phylogenetic trees, in addition to the median parameter estimates we report the 1st and 99th percentiles in the parentheses.
(DOCX)

S3 Table Likelihood ratio tests between models of diversification of bird and bee pollinated lineages of *Ruellia*. Models are defined in Table 1. In bold are likelihood ratio tests where the more complex model was preferred over the simpler model.
(DOCX)

S4 Table Plant fossil calibration priors used in BEAST analysis. The time-calibrated phylogeny (14) was produced using a larger set of Acanthaceae outgroups, to facilitate inclusion of the rich fossil record representative of the family. All outgroups except Acanthopale were later pruned from phylogeny to facilitate diversification analyses. Fossil # refers to information provided in Tripp & McDade (47).
(DOCX)
