# Supplementary figures and images for: Disentangling geographical, biotic, and abiotic drivers of plant diversity in neotropical Ruellia (Acanthaceae)
Source: PLoS One. 2017 May 4;12(5):e0176021. doi: 10.1371/journal.pone.0176021 (PMC5417425; doi:10.1371/journal.pone.0176021)

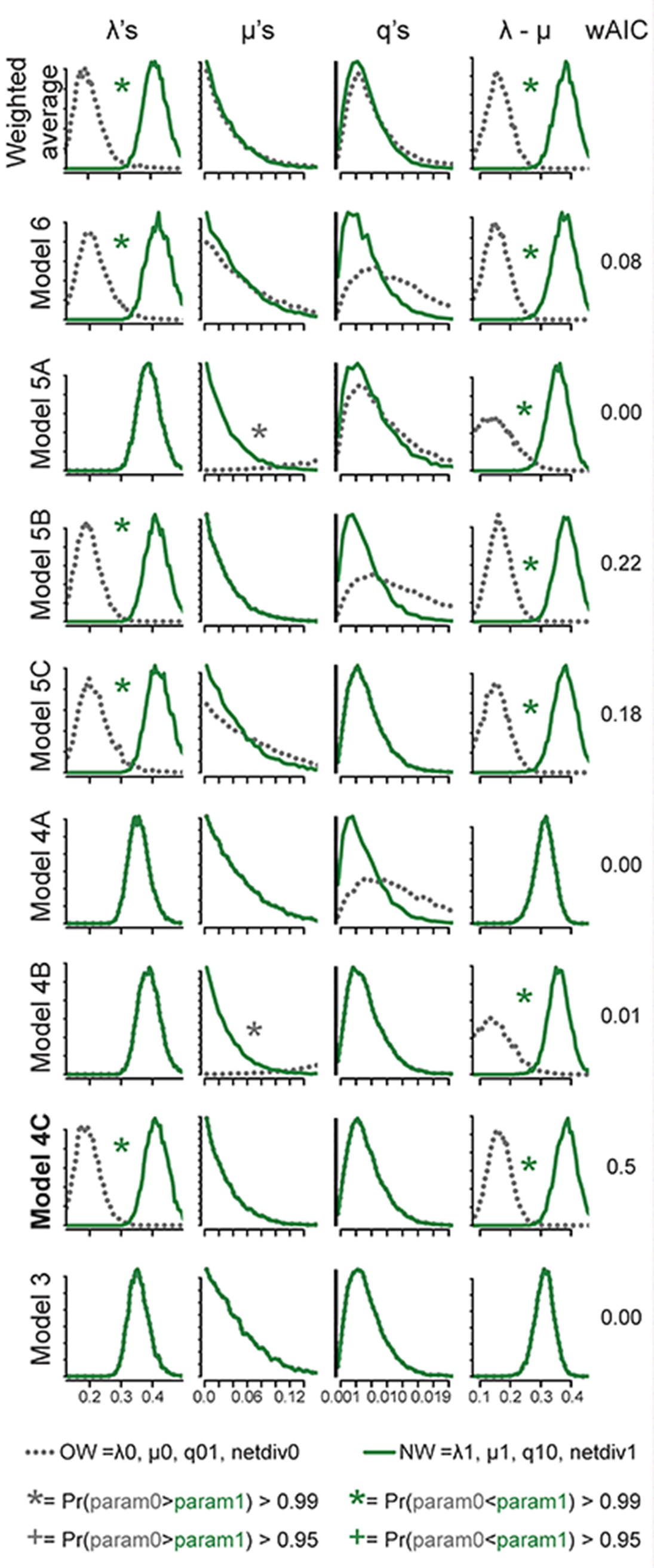

Supplement: S1 Fig — All models including the weighted average model are shown. (TIF) [file pone.0176021.s002.tif]

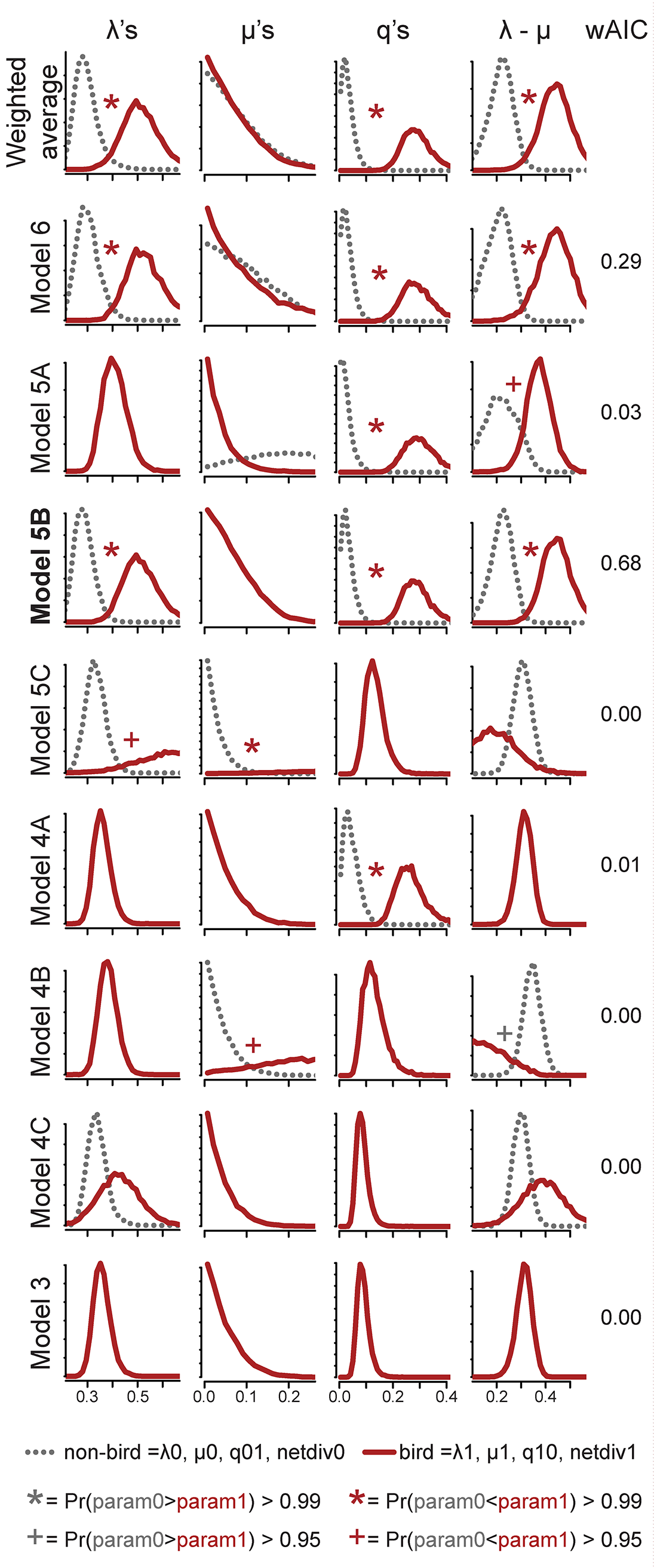

Supplement: S2 Fig — All models including the weighted average model are shown. The best model according to likelihood ratio tests is in bold. (TIF) [file pone.0176021.s003.tif]

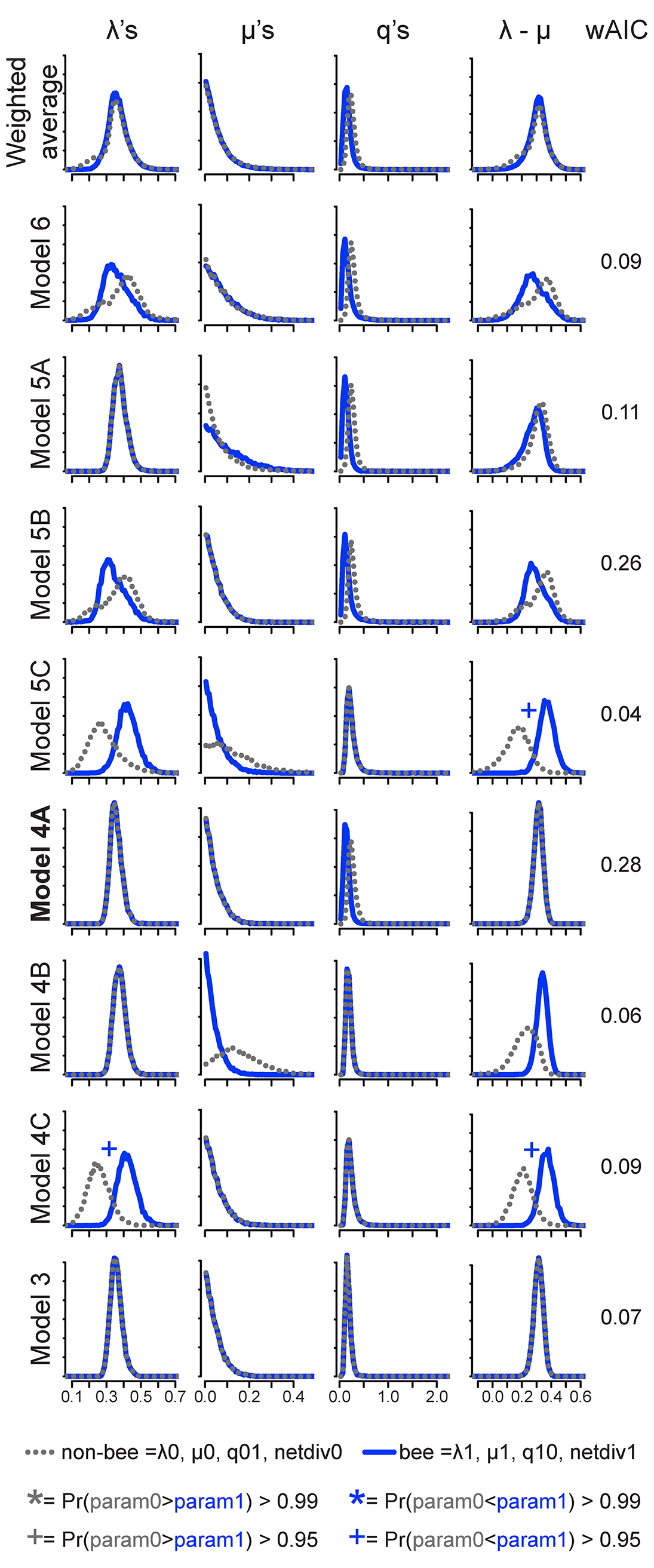

Supplement: S3 Fig — All models including the weighted average model are shown. The best model according to likelihood ratio tests is in bold. (TIF) [file pone.0176021.s004.tif]

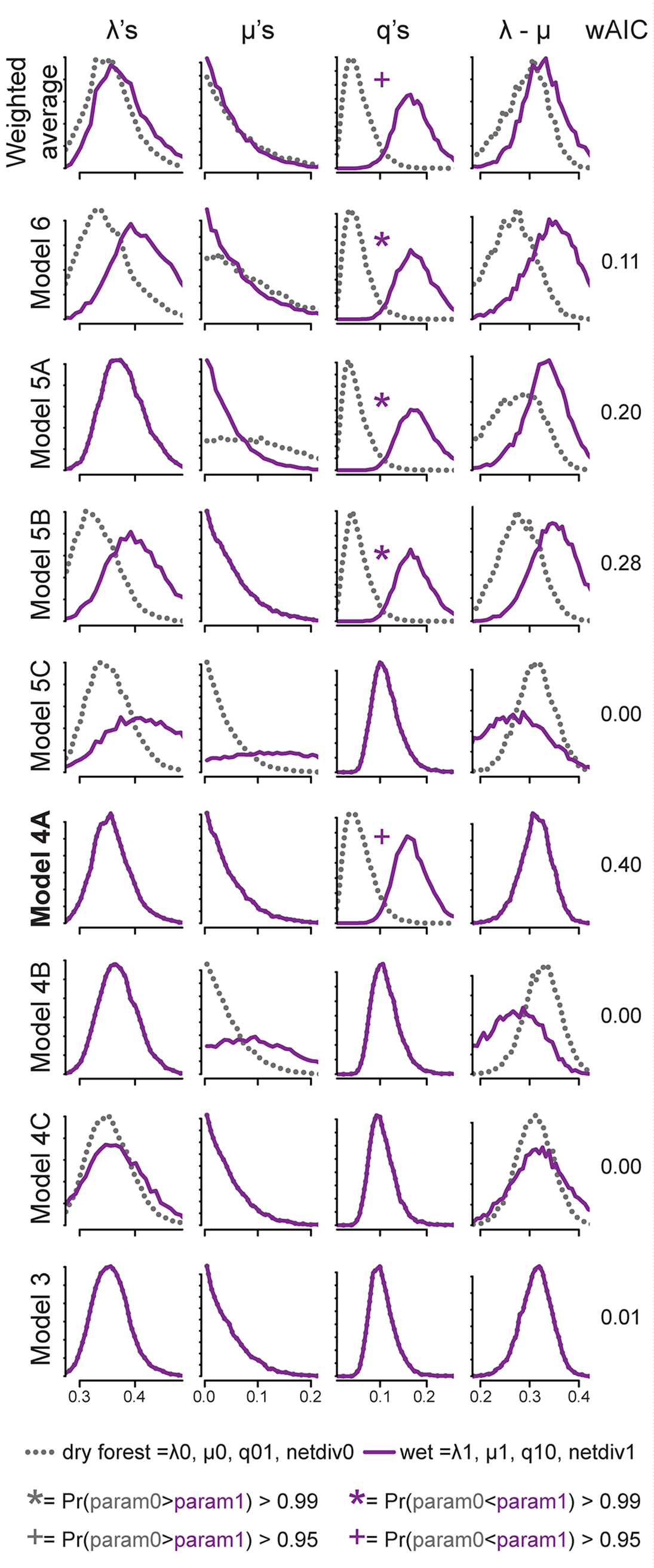

Supplement: S4 Fig — All models including the weighted average model are shown. The best model according to likelihood ratio tests is in bold. (TIF) [file pone.0176021.s005.tif]
