# Supplementary material for: Disentangling geographical, biotic, and abiotic drivers of plant diversity in neotropical Ruellia (Acanthaceae)
Source: PLoS One. 2017 May 4;12(5):e0176021. doi: 10.1371/journal.pone.0176021 (PMC5417425; doi:10.1371/journal.pone.0176021)
Supplement: S1 Table — Models are defined in Table 1. Median parameter estimates are reported with 5th and 95th percentiles given in parentheses. The best models are in bold. (DOCX) [file pone.0176021.s006.docx]

# S1 Table. Diversification models and marginal parameter estimates used to understand the evolution of bee and bird pollination syndromes, habitat shifts, and transitions across continents (Old World to New World) in *Ruellia*. Models are defined in Table 1. Median parameter estimates are reported with 5th and 95th percentiles given in parentheses. The best models are in bold.

|  | **Model** | | **λ0** | | **λ1** | | **μ0** | | **μ1** | | **q01** | | **q10** | | **λ0-μ0** | | **λ1-μ1** | |
| --- | --- | --- | --- | --- | --- | --- | --- | --- | --- | --- | --- | --- | --- | --- | --- | --- | --- | --- |
| OW vs. NW | Weighted Average | | 0.196  (0.137-0.283) | | 0.415  (0.357-0.481) | | 0.0268  (0.0020-0.135) | | 0.0239  (0.0019-0.100) | | 0.00632  (0.0017-0.022) | | 0.00522  (0.0013-0.013) | | 0.16  (0.0905-0.228) | | 0.383  (0.322-0.442) | |
|  | Model 6 | | 0.207  (0.142-0.302) | | 0.42  (0.361-0.493) | | 0.0459  (0.00362-0.18) | | 0.0338  (0.00259-0.13) | | 0.0111  (0.0024-0.032) | | 0.00493  (0.0010-0.014) | | 0.151  (0.0692-0.223) | | 0.378  (0.312-0.441) | |
|  | Model 5A | | 0.388  (0.333-0.451) | | NA | | 0.251  (0.112-0.387) | | 0.0227  (0.0017-0.091) | | 0.0076  (0.0016-0.023) | | 0.00585  (0.0012-0.017) | | 0.138  (0.0254-0.257) | | 0.358  (0.299-0.417) | |
|  | Model 5B | | 0.192  (0.136-0.264) | | 0.413  (0.357-0.48) | | 0.022  (0.0015-0.088) | | NA | | 0.0113  (0.0023-0.032) | | 0.00472  (0.00099-0.01) | | 0.164  (0.103-0.23) | | 0.384  (0.326-0.445) | |
|  | Model 5C | | 0.21  (0.144-0.315) | | 0.42  (0.361-0.494) | | 0.0536  (0.0039-0.195) | | 0.0325  (0.00263-0.13) | | 0.00528  (0.0016-0.013) | | NA | | 0.148  (0.066-0.221) | | 0.379  (0.316-0.442) | |
|  | Model 4A | | 0.355  (0.307-0.417) | | NA | | 0.0346  (0.0027-0.123) | | NA | | 0.0116  (0.0025-0.033) | | 0.00474  (0.001-0.0133) | | 0.314  (0.258-0.363) | | NA | |
|  | Model 4B | | 0.388  (0.336-0.45) | | NA | | 0.254  (0.123-0.389) | | 0.0217  (0.0016-0.088) | | 0.00529  (0.0016-0.013) | | NA | | 0.134  (0.023-0.249) | | 0.359  (0.303-0.417) | |
|  | **Model 4C** | | **0.191**  **(0.136-0.262)** | | **0.413**  **(0.357-0.475)** | | **0.0219**  **(0.0016-0.085)** | | **NA** | | **0.00532**  **(0.0016-0.013)** | | **NA** | | **0.163**  **(0.102-0.229)** | | **0.384**  **(0.326-0.443)** | |
|  | Model 3 | | 0.354  (0.308-0.417) | | NA | | 0.0345  (0.0027-0.122) | | NA | | 0.00536  (0.0016-0.013) | | NA | | 0.313  (0.259-0.363) | | NA | |
| Bird | Weighted Average | 0.289  (0.23-0.378) | | 0.509  (0.398-0.651) | | 0.064  (0.0054-0.214) | | 0.0568  (0.0045-0.201) | | 0.0272  (0.004-0.0739) | | 0.285  (0.209-0.397) | | 0.219  (0.119-0.292) | | 0.44  (0.333-0.549) | |  |
|  | wAvg over 100 Trees | | 0.224  (0.167-0.303) | | 0.457  (0.347-0.614) | | 0.0405  (0.0032-0.152) | | 0.0401  (0.0031-0.164) | | 0.0263  (0.0037-0.076) | | 0.271  (0.186-0.413) | | 0.176  (0.0937-0.246) | | 0.404  (0.301-0.535) | |
|  | Model 6 | | 0.298  (0.233-0.382) | | 0.526  (0.42-0.683) | | 0.0873  (0.00773-0.25) | | 0.07  (0.00542-0.28) | | 0.0265  (0.0036-0.072) | | 0.286  (0.204-0.406) | | 0.206  (0.0933-0.285) | | 0.44  (0.32-0.553) | |
|  | Model 5A | | 0.406  (0.337-0.491) | | NA | | 0.193  (0.0374-0.373) | | 0.0262  (0.0018-0.107) | | 0.0221  (0.0023-0.072) | | 0.295  (0.212-0.401) | | 0.214  (0.0914-0.324) | | 0.372  (0.289-0.459) | |
|  | **Model 5B** | | **0.283**  **(0.228-0.352)** | | **0.507**  **(0.408-0.639)** | | **0.0549**  **(0.0046-0.168)** | | **NA** | | **0.0276**  **(0.0045-0.074)** | | **0.285**  **(0.211-0.394)** | | **0.222**  **(0.135-0.29)** | | **0.444**  **(0.352-0.549)** | |
|  | Model 5C | | 0.33  (0.269-0.4) | | 0.739  (0.45-1.14) | | 0.0192  (0.0015-0.080) | | 0.565  (0.229-0.999) | | 0.129  (0.0826-0.198) | | NA | | 0.305  (0.242-0.368) | | 0.179  (-0.0043-0.35) | |
|  | Model 4A | | 0.356  (0.308-0.419) | | NA | | 0.0353  (0.0027-0.124) | | NA | | 0.0394  (0.0076-0.098) | | 0.26  (0.188-0.372) | | 0.314  (0.258-0.363) | | NA | |
|  | Model 4B | | 0.381  (0.326-0.446) | | NA | | 0.0284  (0.0023-0.107) | | 0.275  (0.0653-0.531) | | 0.124  (0.0747-0.208) | | NA | | 0.345  (0.284-0.404) | | 0.109  (-0.119-0.292) | |
|  | Model 4C | | 0.335  (0.281-0.405) | | 0.425  (0.296-0.564) | | 0.0288  (0.0024-0.111) | | NA | | 0.0824  (0.0555-0.127) | | NA | | 0.299  (0.244-0.355) | | 0.388  (0.246-0.533) | |
|  | Model 3 | | 0.355  (0.307-0.418) | | NA | | 0.0343  (0.0029-0.127) | | NA | | 0.0855  (0.0577-0.13) | | NA | | 0.313  (0.256-0.363) | | NA | |

# S1 Table cont.

|  | **Model** | | **λ0** | | **λ1** | | **μ0** | | **μ1** | | **q01** | | **q10** | | **λ0-μ0** | | **λ1-μ1** | |
| --- | --- | --- | --- | --- | --- | --- | --- | --- | --- | --- | --- | --- | --- | --- | --- | --- | --- | --- |
| Bee | Weighted Average | | 0.359  (0.206-0.479) | | 0.361  (0.276-0.468) | | 0.0434  (0.003-0.193) | | 0.0403  (0.0033-0.163) | | 0.237  (0.138-0.445) | | 0.153  (0.0635-0.333) | | 0.308  (0.122-0.417) | | 0.312  (0.199-0.412) | |
|  | wAvg over 100 Trees | | 0.351  (0.207-0.577) | | 0.266  (0.112-0.384) | | 0.0328  (0.0024-0.155) | | 0.0311  (0.0023-0.133) | | 0.232  (0.117-0.909) | | 0.119  (0.0466-0.483) | | 0.308  (0.142-0.517) | | 0.226  (0.0587-0.339) | |
|  | Model 6 | | 0.403  (0.192-0.548) | | 0.351  (0.254-0.49) | | 0.0636  (0.0054-0.246) | | 0.0628  (0.005-0.22) | | 0.259  (0.158-0.583) | | 0.137  (0.0528-0.413) | | 0.33  (0.0709-0.457) | | 0.274  (0.137-0.426) | |
|  | Model 5A | | 0.376  (0.321-0.449) | | NA | | 0.0459  (0.004-0.212) | | 0.0799  (0.006-0.276) | | 0.25  (0.154-0.414) | | 0.126  (0.0505-0.259) | | 0.321  (0.175-0.406) | | 0.287  (0.144-0.37) | |
|  | Model 5B | | 0.386  (0.19-0.513) | | 0.333  (0.25-0.467) | | 0.0388  (0.003-0.136) | | NA | | 0.256  (0.164-0.578) | | 0.137  (0.0589-0.398) | | 0.341  (0.139-0.453) | | 0.286  (0.187-0.418) | |
|  | Model 5C | | 0.276  (0.16-0.46) | | 0.421  (0.332-0.524) | | 0.109  (0.0113-0.32) | | 0.0392  (0.0032-0.147) | | 0.214  (0.13-0.416) | | NA | | 0.168  (-0.01-0.297) | | 0.37  (0.284-0.461) | |
|  | **Model 4A** | | **0.353**  **(0.306-0.416)** | | **NA** | | **0.033**  **(0.003-0.122)** | | **NA** | | **0.246**  **(0.159-0.419)** | | **0.139**  **(0.0654-0.262)** | | **0.313**  **(0.258-0.363)** | | **NA** | |
|  | Model 4B | | 0.375  (0.322-0.439) | | NA | | 0.147  (0.0247-0.328) | | 0.0291  (0.002-0.109) | | 0.184  (0.118-0.297) | | NA | | 0.227  (0.0767-0.333) | | 0.339  (0.274-0.398) | |
|  | Model 4C | | 0.253  (0.153-0.37) | | 0.418  (0.337-0.516) | | 0.0397  (0.0032-0.138) | | NA | | 0.207  (0.124-0.393) | | NA | | 0.205  (0.0881-0.32) | | 0.369  (0.288-0.456) | |
|  | Model 3 | | 0.354  (0.306-0.416) | | NA | | 0.0336  (0.003-0.121) | | NA | | 0.169  (0.108-0.277) | | NA | | 0.314  (0.259-0.363) | | NA | |
| Habitat | Weighted Average | 0.351  (0.284-0.428) | | 0.373  (0.312-0.473) | | 0.0463  (0.0035-0.189) | | 0.0367  (0.0029-0.144) | | 0.0487  (0.0172-0.11) | | 0.169  (0.114-0.248) | | 0.294  (0.196-0.357) | | 0.328  (0.256-0.412) | |  |
|  | Model 6 | | 0.341  (0.274-0.438) | | 0.412  (0.33-0.523) | | 0.0716  (0.0069-0.226) | | 0.0508  (0.0038-0.204) | | 0.0474  (0.0171-0.105) | | 0.176  (0.118-0.259) | | 0.262  (0.161-0.339) | | 0.35  (0.252-0.439) | |
|  | Model 5A | | 0.375  (0.321-0.448) | | NA | | 0.102  (0.0109-0.259) | | 0.0342  (0.0028-0.131) | | 0.0433  (0.0156-0.101) | | 0.178  (0.121-0.258) | | 0.271  (0.159-0.352) | | 0.334  (0.253-0.406) | |
|  | Model 5B | | 0.326  (0.265-0.399) | | 0.399  (0.321-0.497) | | 0.0387  (0.00297-0.14) | | NA | | 0.0475  (0.0177-0.101) | | 0.171  (0.118-0.246) | | 0.279  (0.205-0.35) | | 0.351  (0.274-0.434) | |
|  | Model 5C | | 0.35  (0.291-0.424) | | 0.428  (0.314-0.603) | | 0.0297  (0.0025-0.113) | | 0.159  (0.0199-0.388) | | 0.11  (0.0723-0.169) | | NA | | 0.312  (0.249-0.376) | | 0.266  (0.139-0.38) | |
|  | **Model 4A** | | **0.355**  **(0.306-0.42)** | | **NA** | | **0.0336**  **(0.0028-0.128)** | | **NA** | | **0.0513**  **(0.0178-0.11)** | | **0.166**  **(0.114-0.239)** | | **0.314**  **(0.257-0.363)** | | **NA** | |
|  | Model 4B | | 0.368  (0.317-0.431) | | NA | | 0.0348  (0.0028-0.123) | | 0.1  (0.0116-0.249) | | 0.108  (0.0724-0.165) | | NA | | 0.326  (0.262-0.384) | | 0.265  (0.143-0.352) | |
|  | Model 4C | | 0.351  (0.292-0.425) | | 0.363  (0.277-0.46) | | 0.0337  (0.00263-0.12) | | NA | | 0.101  (0.0685-0.154) | | NA | | 0.31  (0.247-0.372) | | 0.32  (0.229-0.418) | |
|  | Model 3 | | 0.355  (0.306-0.415) | | NA | | 0.0335  (0.00255-0.12) | | NA | | 0.101  (0.0688-0.153) | | NA | | 0.314  (0.259-0.363) | | NA | |
