# Supplementary material for: Disentangling geographical, biotic, and abiotic drivers of plant diversity in neotropical Ruellia (Acanthaceae)
Source: PLoS One. 2017 May 4;12(5):e0176021. doi: 10.1371/journal.pone.0176021 (PMC5417425; doi:10.1371/journal.pone.0176021)
Supplement: S2 Table — Models are defined in Table 1. Asterisks indicate significant values either less than 0.05 or greater than 0.95. NA’s indicate comparisons of parameters that were set to be equal in the model. The best models are shown in bold. For analyses averaged over 100 phylogenetic trees, in addition to the median parameter estimates we report the 1st and 99th percentiles in the parentheses. (DOCX) [file pone.0176021.s007.docx]

# S2 Table. Probabilities that the marginal distribution of one parameter is less than another parameter. Models are defined in Table 1. Asterisks indicate significant values either less than 0.05 or greater than 0.95. NA’s indicate comparisons of parameters that were set to be equal in the model. The best models are shown in bold. For analyses averaged over 100 phylogenetic trees, in addition to the median parameter estimates we report the 1st and 99th percentiles in the parentheses.

|  | **Model** | **Pr(λ0 < λ1)** | **Pr(μ0 < μ1)** | **Pr(q01 < q10)** | **Pr(λ0-μ0 < λ1-μ1)** |
| --- | --- | --- | --- | --- | --- |
| OW vs NW | Weighted Average | 0.999* | 0.470 | 0.402 | 1.000* |
|  | Model 6 | 0.996* | 0.419 | 0.210 | 0.999* |
|  | Model 5A | 0.494 | 0.009* | 0.385 | 1.000* |
|  | Model 5B | 0.999* | 0.516 | 0.219 | 0.999* |
|  | Model 5C | 0.989* | 0.357 | 0.501 | 1.000* |
|  | Model 4A | 0.478 | 0.528 | 0.191 | 0.497 |
|  | Model 4B | 0.505 | 0.006* | 0.501 | 0.999* |
|  | **Model 4C** | **1.000*** | **0.509** | **0.521** | **1.000*** |
|  | Model 3 | 0.483 | 0.512 | 0.509 | 0.485 |
| Bird | Weighted Average | 0.996* | 0.480 | 1.000* | 0.997* |
|  | wAvg over 100 trees | 0.996*  (0.997-1) | 0.505  (0.446-0.546) | 0.999*  (0.996-1) | 0.999*  (0.997-1) |
|  | Model 6 | 0.999* | 0.445 | 1.000* | 0.996* |
|  | Model 5A | 0.488 | 0.062 | 1.000* | 0.970* |
|  | **Model 5B** | **0.997*** | **0.462** | **1.000*** | **1.000*** |
|  | Model 5C | 0.988* | 0.997* | 0.524 | 0.119 |
|  | Model 4A | 0.493 | 0.506 | 0.999* | 0.473 |
|  | Model 4B | 0.511 | 0.972* | 0.511 | 0.019* |
|  | Model 4C | 0.831 | 0.502 | 0.481 | 0.846 |
|  | Model 3 | 0.505 | 0.552 | 0.493 | 0.486 |
| Bee | Weighted Average | 0.543 | 0.470 | 0.201 | 0.532 |
|  | wAvg over 100 trees | 0.206  (0.002-0.65) | 0.485  (0.406-0.539) | 0.192  (0.018-0.395) | 0.229  (0.001-0.681) |
|  | Model 6 | 0.370 | 0.449 | 0.190 | 0.368 |
|  | Model 5A | 0.479 | 0.614 | 0.094 | 0.345 |
|  | Model 5B | 0.381 | 0.510 | 0.147 | 0.354 |
|  | Model 5C | 0.883 | 0.237 | 0.481 | 0.987* |
|  | **Model 4A** | **0.475** | **0.494** | **0.128** | **0.502** |
|  | Model 4B | 0.500 | 0.104 | 0.508 | 0.913 |
|  | Model 4C | 0.968* | 0.482 | 0.444 | 0.974* |
|  | Model 3 | 0.495 | 0.485 | 0.489 | 0.486 |
| Habitat | Weighted average | 0.671 | 0.437 | 0.985* | 0.726 |
|  | Model 6 | 0.846 | 0.412 | 0.993* | 0.882 |
|  | Model 5A | 0.476 | 0.213 | 0.996* | 0.828 |
|  | Model 5B | 0.870 | 0.522 | 0.995* | 0.856 |
|  | Model 5C | 0.820 | 0.899 | 0.500 | 0.294 |
|  | **Model 4A** | **0.532** | **0.513** | **0.988*** | **0.486** |
|  | Model 4B | 0.502 | 0.804 | 0.487 | 0.202 |
|  | Model 4C | 0.554 | 0.548 | 0.478 | 0.561 |
|  | Model 3 | 0.498 | 0.493 | 0.498 | 0.513 |
