# Supplementary material for: Disentangling geographical, biotic, and abiotic drivers of plant diversity in neotropical Ruellia (Acanthaceae)
Source: PLoS One. 2017 May 4;12(5):e0176021. doi: 10.1371/journal.pone.0176021 (PMC5417425; doi:10.1371/journal.pone.0176021)
Supplement: S3 Table — Models are defined in Table 1. In bold are likelihood ratio tests where the more complex model was preferred over the simpler model. (DOCX) [file pone.0176021.s008.docx]

# S3 Table. Likelihood ratio tests between models of diversification of bird and bee pollinated lineages of *Ruellia*. Models are defined in Table 1. In bold are likelihood ratio tests where the more complex model was preferred over the simpler model.

|  | **ModA** | **ModB** | **No. Par_A_** | **No. Par_B_** | **lnLik_A_** | **lnLik_B_** | **AIC_A_** | **AIC_B_** | **ChiSq** | **Prob** |
| --- | --- | --- | --- | --- | --- | --- | --- | --- | --- | --- |
| OW vs NW | **6** | **5A** | **6** | **5** | **-364.55** | **-368.98** | **741.09** | **747.96** | **8.86** | **0** |
|  | 6 | 5B | 6 | 5 | -364.55 | -364.55 | 741.09 | 739.09 | 0 | 1 |
|  | 6 | 5C | 6 | 5 | -364.55 | -364.75 | 741.09 | 739.49 | 0.4 | 0.53 |
|  | **5A** | **4A** | **5** | **4** | **-368.98** | **-373.43** | **747.96** | **754.86** | **8.9** | **0** |
|  | **5B** | **4A** | **5** | **4** | **-364.55** | **-373.43** | **739.09** | **754.86** | **17.76** | **0** |
|  | 5A | 4B | 5 | 4 | -368.98 | -368.98 | 747.96 | 745.97 | 0.01 | 0.91 |
|  | **5C** | **4B** | **5** | **4** | **-364.75** | **-368.98** | **739.49** | **745.97** | **8.48** | **0** |
|  | 5B | 4C | 5 | 4 | -364.55 | -364.75 | 739.09 | 737.49 | 0.4 | 0.53 |
|  | 5C | 4C | 5 | 4 | -364.75 | -364.75 | 739.49 | 737.49 | 0 | 1 |
|  | 4A | 3 | 4 | 3 | -373.43 | -373.63 | 754.86 | 753.25 | 0.4 | 0.53 |
|  | **4B** | **3** | **4** | **3** | **-368.98** | **-373.63** | **745.97** | **753.25** | **9.29** | **0** |
|  | **4C** | **3** | **4** | **3** | **-364.75** | **-373.63** | **737.49** | **753.25** | **17.76** | **0** |
|  | **6** | **4A** | **6** | **4** | **-364.55** | **-373.43** | **741.09** | **754.86** | **17.76** | **0** |
|  | **6** | **4B** | **6** | **4** | **-364.55** | **-368.98** | **741.09** | **745.97** | **8.87** | **0.01** |
|  | 6 | 4C | 6 | 4 | -364.55 | -364.75 | 741.09 | 737.49 | 0.4 | 0.82 |
|  | **6** | **3** | **6** | **3** | **-364.55** | **-373.63** | **741.09** | **753.25** | **18.16** | **0** |
|  | **5A** | **3** | **5** | **3** | **-368.98** | **-373.63** | **747.96** | **753.25** | **9.3** | **0.01** |
|  | **5B** | **3** | **5** | **3** | **-364.55** | **-373.63** | **739.09** | **753.25** | **18.16** | **0** |
|  | **5C** | **3** | **5** | **3** | **-364.75** | **-373.63** | **739.49** | **753.25** | **17.76** | **0** |
| Bird | **6** | **5A** | **6** | **5** | **-435.53** | **-438.94** | **883.07** | **887.88** | **6.82** | **0.01** |
|  | 6 | 5B | 6 | 5 | -435.53 | -435.68 | 883.07 | 881.36 | 0.29 | 0.59 |
|  | **6** | **5C** | **6** | **5** | **-435.53** | **-447.41** | **883.07** | **904.81** | **23.75** | **0** |
|  | 5A | 4A | 5 | 4 | -438.94 | -440.87 | 887.88 | 889.74 | 3.86 | 0.05 |
|  | **5B** | **4A** | **5** | **4** | **-435.68** | **-440.87** | **881.36** | **889.74** | **10.38** | **0** |
|  | **5A** | **4B** | **5** | **4** | **-438.94** | **-451.1** | **887.88** | **910.21** | **24.33** | **0** |
|  | **5C** | **4B** | **5** | **4** | **-447.41** | **-451.1** | **904.81** | **910.21** | **7.4** | **0.01** |
|  | **5B** | **4C** | **5** | **4** | **-435.68** | **-451.83** | **881.36** | **911.66** | **32.3** | **0** |
|  | **5C** | **4C** | **5** | **4** | **-447.41** | **-451.83** | **904.81** | **911.66** | **8.85** | **0** |
|  | **4A** | **3** | **4** | **3** | **-440.87** | **-452.75** | **889.74** | **911.5** | **23.76** | **0** |
|  | 4B | 3 | 4 | 3 | -451.1 | -452.75 | 910.21 | 911.5 | 3.29 | 0.07 |
|  | 4C | 3 | 4 | 3 | -451.83 | -452.75 | 911.66 | 911.5 | 1.84 | 0.18 |
|  | **6** | **4A** | **6** | **4** | **-435.53** | **-440.87** | **883.07** | **889.74** | **10.67** | **0** |
|  | **6** | **4B** | **6** | **4** | **-435.53** | **-451.1** | **883.07** | **910.21** | **31.14** | **0** |
|  | **6** | **4C** | **6** | **4** | **-435.53** | **-451.83** | **883.07** | **911.66** | **32.59** | **0** |
|  | **6** | **3** | **6** | **3** | **-435.53** | **-452.75** | **883.07** | **911.5** | **34.43** | **0** |
|  | **5A** | **3** | **5** | **3** | **-438.94** | **-452.75** | **887.88** | **911.5** | **27.61** | **0** |
|  | **5B** | **3** | **5** | **3** | **-435.68** | **-452.75** | **881.36** | **911.5** | **34.14** | **0** |
|  | **5C** | **3** | **5** | **3** | **-447.41** | **-452.75** | **904.81** | **911.5** | **10.68** | **0** |
| Bee | 6 | 5A | 6 | 5 | -464.99 | -465.86 | 941.98 | 941.71 | 1.74 | 0.19 |
|  | 6 | 5B | 6 | 5 | -464.99 | -464.99 | 941.98 | 939.98 | 0 | 1 |
|  | 6 | 5C | 6 | 5 | -464.99 | -466.94 | 941.98 | 943.88 | 3.9 | 0.05 |
|  | 5A | 4A | 5 | 4 | -465.86 | -465.89 | 941.71 | 939.78 | 0.07 | 0.79 |
|  | 5B | 4A | 5 | 4 | -464.99 | -465.89 | 939.98 | 939.78 | 1.8 | 0.18 |
|  | 5A | 4B | 5 | 4 | -465.86 | -467.51 | 941.71 | 943.02 | 3.31 | 0.07 |
|  | 5C | 4B | 5 | 4 | -466.94 | -467.51 | 943.88 | 943.02 | 1.14 | 0.29 |
|  | **5B** | **4C** | **5** | **4** | **-464.99** | **-467.01** | **939.98** | **942.01** | **4.04** | **0.04** |
|  | 5C | 4C | 5 | 4 | -466.94 | -467.01 | 943.88 | 942.01 | 0.13 | 0.71 |
|  | **4A** | **3** | **4** | **3** | **-465.89** | **-468.33** | **939.78** | **942.66** | **4.88** | **0.03** |
|  | 4B | 3 | 4 | 3 | -467.51 | -468.33 | 943.02 | 942.66 | 1.65 | 0.2 |
|  | 4C | 3 | 4 | 3 | -467.01 | -468.33 | 942.01 | 942.66 | 2.65 | 0.1 |
|  | 6 | 4A | 6 | 4 | -464.99 | -465.89 | 941.98 | 939.78 | 1.8 | 0.41 |
|  | 6 | 4B | 6 | 4 | -464.99 | -467.51 | 941.98 | 943.02 | 5.04 | 0.08 |
|  | 6 | 4C | 6 | 4 | -464.99 | -467.01 | 941.98 | 942.01 | 4.04 | 0.13 |
|  | 6 | 3 | 6 | 3 | -464.99 | -468.33 | 941.98 | 942.66 | 6.69 | 0.08 |
|  | 5A | 3 | 5 | 3 | -465.86 | -468.33 | 941.71 | 942.66 | 4.95 | 0.08 |
|  | **5B** | **3** | **5** | **3** | **-464.99** | **-468.33** | **939.98** | **942.66** | **6.69** | **0.04** |
|  | 5C | 3 | 5 | 3 | -466.94 | -468.33 | 943.88 | 942.66 | 2.79 | 0.25 |
| Habitat | 6 | 5A | 6 | 5 | -451.29 | -451.66 | 914.57 | 913.32 | 0.74 | 0.39 |
|  | 6 | 5B | 6 | 5 | -451.29 | -451.3 | 914.57 | 912.59 | 0.02 | 0.89 |
|  | **6** | **5C** | **6** | **5** | **-451.29** | **-456.19** | **914.57** | **922.38** | **9.81** | **0** |
|  | 5A | 4A | 5 | 4 | -451.66 | -451.96 | 913.32 | 911.92 | 0.61 | 0.44 |
|  | 5B | 4A | 5 | 4 | -451.3 | -451.96 | 912.59 | 911.92 | 1.33 | 0.25 |
|  | **5A** | **4B** | **5** | **4** | **-451.66** | **-456.55** | **913.32** | **921.1** | **9.78** | **0** |
|  | 5C | 4B | 5 | 4 | -456.19 | -456.55 | 922.38 | 921.1 | 0.72 | 0.4 |
|  | **5B** | **4C** | **5** | **4** | **-451.3** | **-456.61** | **912.59** | **921.23** | **10.64** | **0** |
|  | 5C | 4C | 5 | 4 | -456.19 | -456.61 | 922.38 | 921.23 | 0.85 | 0.36 |
|  | **4A** | **3** | **4** | **3** | **-451.96** | **-456.67** | **911.92** | **919.35** | **9.43** | **0** |
|  | 4B | 3 | 4 | 3 | -456.55 | -456.67 | 921.1 | 919.35 | 0.25 | 0.62 |
|  | 4C | 3 | 4 | 3 | -456.61 | -456.67 | 921.23 | 919.35 | 0.12 | 0.73 |
|  | 6 | 4A | 6 | 4 | -451.29 | -451.96 | 914.57 | 911.92 | 1.35 | 0.51 |
|  | **6** | **4B** | **6** | **4** | **-451.29** | **-456.55** | **914.57** | **921.1** | **10.52** | **0.01** |
|  | **6** | **4C** | **6** | **4** | **-451.29** | **-456.61** | **914.57** | **921.23** | **10.66** | **0** |
|  | **6** | **3** | **6** | **3** | **-451.29** | **-456.67** | **914.57** | **919.35** | **10.78** | **0.01** |
|  | **5A** | **3** | **5** | **3** | **-451.66** | **-456.67** | **913.32** | **919.35** | **10.03** | **0.01** |
|  | **5B** | **3** | **5** | **3** | **-451.3** | **-456.67** | **912.59** | **919.35** | **10.76** | **0** |
|  | 5C | 3 | 5 | 3 | -456.19 | -456.67 | 922.38 | 919.35 | 0.97 | 0.62 |
