# Supplementary material for: Disentangling geographical, biotic, and abiotic drivers of plant diversity in neotropical Ruellia (Acanthaceae)
Source: PLoS One. 2017 May 4;12(5):e0176021. doi: 10.1371/journal.pone.0176021 (PMC5417425; doi:10.1371/journal.pone.0176021)
Supplement: S4 Table — The time-calibrated phylogeny (14) was produced using a larger set of Acanthaceae outgroups, to facilitate inclusion of the rich fossil record representative of the family. All outgroups except Acanthopale were later pruned from phylogeny to facilitate diversification analyses. Fossil # refers to information provided in Tripp & McDade (47). (DOCX) [file pone.0176021.s009.docx]

# S4 Table. Plant fossil calibration priors used in BEAST analysis. The time-calibrated phylogeny (14) was produced using a larger set of Acanthaceae outgroups, to facilitate inclusion of the rich fossil record representative of the family. All outgroups except Acanthopale were later pruned from phylogeny to facilitate diversification analyses. Fossil # refers to information provided in Tripp & McDade (47).

| **Taxon Set** | **Fossil** | **Taxa Constrained** | **Age** | **Zero Offset** | **Log (Stdev)** | **Mean** | **5% quantile** | **95% quantile** |
| --- | --- | --- | --- | --- | --- | --- | --- | --- |
| TS 1 | N/A | All Acanthaceae monophyletic | None designated (used tree prior) | N/A | N/A | N/A | N/A | N/A |
| TS 2 | #1 | *Avicennia* (2 species) | mid Bartonian (~39.4-38.3 Ma) | 38.3 | 0.6 | 0.5 | 38.46 | 39.42 |
| TS 3 | #11 | *Acanthopsis* + *Acanthus* | Early-Mid Oligocene (~33.7-28.8 Ma) | 28.8 | 1.1 | 1.5 | 28.9 | 33.8 |
| TS 4 | #15 | Barlerieae (2 taxa) | Miocene (~23.8-5.3 Ma) | 5.3 | 1.1 | 5.5 | 5.8 | 23.6 |
| TS 5 | #30 | *Dicliptera* + *Rhinacanthus* | Lower Miocene (~23.8-14.6 Ma) | 14.6 | 1.3 | 2.5 | 14.7 | 23.7 |
| TS 6 | #36 | pseudocolpate Ruellieae excluding Trichantherinae (8 taxa) | Upper Miocene (~14.55-5.3 Ma) | 5.3 | 1.4 | 2.5 | 5.4 | 14.7 |
| TS 7 | #37 | Petalidiinae + Mimulopsiinae (5 taxa) | Mio-Pliocene (~23.8-1.8 Ma) | 1.8 | 1.3 | 6 | 2.1 | 23.7 |
| TS 8 | #46 | Trichantherinae excluding Louteridium (3 taxa) | 10-12 Ma | 10 | 0.5 | 1 | 10.4 | 12.0 |
